# Improving the Effectiveness of Multi-Agent Cooperation for Green Manufacturing in China: A Theoretical Framework to Measure the Performance of Green Technology Innovation

**DOI:** 10.3390/ijerph17093211

**Published:** 2020-05-05

**Authors:** Shi Yin, Nan Zhang, Baizhou Li

**Affiliations:** 1 College of Economics and Management, Hebei Agricultural University, Baoding 071000, China; 2 School of Economics and Management, Harbin Engineering University, Harbin 150001, China

**Keywords:** green technology innovation, multi-agent cooperation, manufacturing enterprises, performance evaluation

## Abstract

A green manufacturing system is an important tool to realize green transformation of the manufacturing industry. The systematicness of green technology innovation as the key foundation of green manufacturing supports the entire huge green manufacturing system. In order to improve the effectiveness of multi-agent cooperation, it is necessary to analyze a series of green technology innovation achievements of manufacturing enterprises under multi-agent cooperation. First of all, inter-indicator correlation analysis and exploratory factor analysis were used to construct the evaluation index system of the green technology innovation performance of manufacturing enterprises under multi-agent cooperation. Then, a secondary combined evaluation model was constructed based on the evaluation conclusions. Finally, a theoretical framework was constructed to measure the performance of the green technology innovation of manufacturing enterprises under multi-agent cooperation. The results of this study are as follows: The evaluation index system of the green technology innovation performance of manufacturing enterprises under multi-agent cooperation is composed of the technology output, economic output, and social effect of green technology innovation. The key factors that influence the green technology innovation performance of manufacturing enterprises under multi-agent cooperation are the proportion of green technology transformation in traditional technology, the number of papers published jointly by multi-agent cooperation, the user acceptance of green technology products, and the degree of improvement of public environmental preference and consciousness. A fusion of technology of subjective and objective methods is an effective evaluation technique and can be applied to evaluate the performance of green technology innovation. The secondary combined evaluation combines the evaluation conclusions obtained by each single evaluation method in a certain form.

## 1. Introduction

Global climate change is an indisputable phenomenon and has become one of the greatest challenges facing human development in the 21st century [1]. The manufacturing industry is an important force in promoting industrialization, as well as a major source of pollutant emissions [2]. At present, China’s manufacturing industry is facing a serious waste of resources. The growth rate of the manufacturing industry has dropped significantly in the face of a series of prominent contradictions such as excess capacity, an imbalance between supply and demand, and slow transformation. In the past three years, the average discharge of waste water per unit of manufacturing output value was more than 125,000 tons/100 million yuan—far higher than that of developed countries. Furthermore, chlorine dioxide emissions, solid waste emissions, and soot and dust emissions are all below the level of 0.05 tons/100 yuan, indicating that the green governance power of the manufacturing industry is still insufficient [3]. According to the target set in the “Made in China 2025,” the above three items are to be reduced by 34%, 40%, and 41%, respectively. From the perspective of structural optimization, there are still numerous low-end and inefficient links in China’s manufacturing industry [4]. In 2018, the added value of China’s high-tech manufacturing industry accounted for only 13.9% of the industrial added value above the designated size, and thus still has huge room for improvement in the industrial structure [5]. From the perspective of innovation ability, manufacturing technology’s innovation ability is still not strong and not enough to support industrial development. In 2017, the research and development (R&D) intensity of China’s manufacturing industry above the designated size was only 1.14%. China’s Huawei is the only one of the top 50 companies in the world to invest in R&D [6]. From the perspective of green development, compared to developed countries, the utilization efficiency of energy and resources is still low. The carrying capacity of resources and environment is close to the upper limit. At the same time, the original growth model in China, characterized by quantity, scale, and speed, has failed to meet the requirements of the new norm of economic development [7]. Green development is an important opportunity for the development of new industries such as energy conservation and environmental protection [8]. Therefore, the key to solving this problem is to promote green manufacturing based on the deep integration of manufacturing industry and information technology. 

The “Made in China 2025” action plan emphasizes the importance of green development in the transformation of the manufacturing industry [9]. The government should intensify the R&D of advanced energy-saving and environmental protection technologies, and should build an efficient, clean, low-carbon, and circular green manufacturing system. The 2019 Government Work Report further pointed out that high-quality development of manufacturing should strengthen the industrial base and the technological innovation capacity. Fully implementing green manufacturing is not only one of the important strategic tasks, but also the only way to develop into a manufacturing power. Green manufacturing runs through the whole process, from R&D to sale, and needs to be supported by green technology. A single green technology innovation in a certain link cannot support a huge system of green manufacturing, and thus systematic green technology innovation is necessary [10]. Green technology innovation has become the core mechanism to reconcile the sharp contradiction between environmental protection and economic development, and is also key to the green development of China’s manufacturing industry. A green technology innovation system includes the innovation subject, the innovation environment, the innovation resources, the innovation infrastructure, and various other elements. The system is a top-down hierarchical system that requires the participation of a whole society [11]. A well-run system should focus on the service innovation subject and should contribute to the innovation resources, the innovation environment, and the innovation infrastructure. At the same time, a policy support system should be systematically constructed to promote the vigorous development of green technology innovation [12].

China’s 13th Five-Year Plan for technology innovation pointed out that the close combination of government, industry, university, and research institutions can improve the level of technology innovation. The report to the 19th national congress of the Communist Party of China (CPC) commented that a technology innovation system should be established with enterprises as the main body and the market as the orientation, alongside the deep integration of industry, university, and research institutions [13]. In the report, it was further mentioned that the integrated innovation mechanism of industry, university, and research institutions with enterprises as the main body should be improved. A system of green technology innovation is a multi-agent cooperation system that promotes high-quality development of the manufacturing industry [14]. Hence, government, industry, university, and research institutions and consumers should be closely linked. The formation of a multi-agent cooperation is of great significance to improve the mechanism of green technology innovation with manufacturing enterprises as the main body [15].

The performance evaluation of the green technology innovation of manufacturing enterprises under multi-agent cooperation is to analyze the innovation achievements obtained through a series of green technology innovation activities [16]. The purpose of this evaluation is to understand the efficiency of the green technology innovation of manufacturing enterprises under multi-agent cooperation. The performance evaluation is a comprehensive evaluation of whether the green technology innovation activities carried out by manufacturing enterprises under multi-agent cooperation have produced benefits. The stage of a green technology innovation activity determines the hierarchy of the evaluation index system of the green technology innovation performance of manufacturing enterprises under multi-agent cooperation [17]. The successful development of new products and process innovation related to green technology based on multi-agent cooperation can bring huge green economic benefits to manufacturing enterprises and can change the use of production factors and the social environment [18].

**Figure 1:**
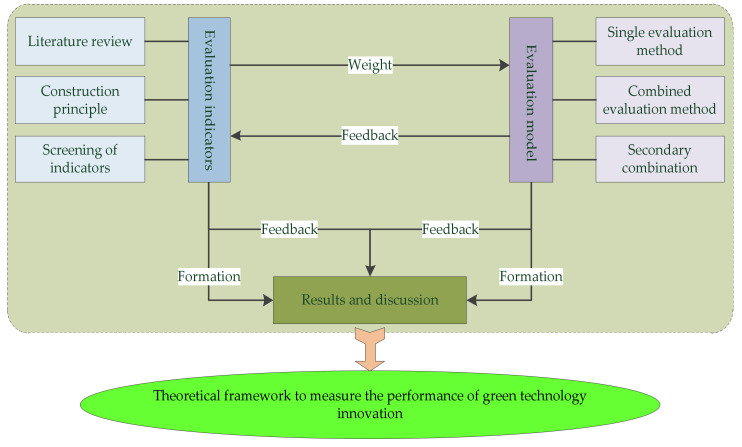
The analytical framework of green technology innovation performance evaluation of manufacturing enterprises under multi-agent cooperation.

Many scholars have studied the aspects related to industry, university, and research institutions [8,13]. However, there are fewer studies on the green technology innovation process of enterprises under multi-agent cooperation [18,19,20,21,22,23,24,25,26,27,28,29,30,31,32,33,34,35,36,37,38]. The existing studies related to the index system pay more attention to the combination of quantitative and qualitative indices [39,40,41,42,43,44,45,46,47,48,49,50,51,52,53,54,55,56,57,58,59,60,61,62,63,64,65,66]. However, neither the evaluation index system of green innovation performance nor the evaluation index system of multi-agent cooperation performance has been studied from the perspective of a theoretical framework. As a result, the green technology innovation activities of manufacturing enterprises under multi-agent cooperation lack the corresponding theoretical support, which is not conducive to the development of green technology innovation activities. The existing studies hinder the improvement of green technology innovation capability of manufacturing enterprises. In terms of evaluation methods, data envelopment analysis (DEA) model, combination weight, analytical hierarchy process (AHP), entropy weight method, principal component analysis, and other single evaluation methods have all been adopted in most of these studies. However, the use of such a wide variety of methods leads to a lack of consistency in the evaluation results. Therefore, this study mainly constructs a theoretical framework to deal with the evaluation of the green technology innovation performance of manufacturing enterprises under multi-agent cooperation. The main purpose of this study is as shown in Figure 1. 

As can be seen in Figure 1, the analytical framework includes the evaluation index system and the evaluation model. Firstly, inter-indicator correlation analysis and exploratory factor analysis were used to construct the evaluation index system of the green technology innovation performance of manufacturing enterprises under multi-agent cooperation. Then, a secondary combined evaluation model was constructed based on the evaluation conclusions. Finally, a theoretical framework was constructed to measure the performance of the green technology innovation of manufacturing enterprises under multi-agent cooperation. The rest of this study is structured as follows. Section 2 outlines the literature review. The evaluation index system is shown in Section 3. In Section 4, the secondary combined evaluation model is constructed. Section 5 constructs the theoretical framework. Finally, the research conclusions and future research directions are shown in Section 6.

## 2. Literature Review

In this study, the analytical framework includes the evaluation index system and the evaluation model. Keywords such as “multi-agent cooperation,” “cooperative innovation,” “green technology innovation,” and “innovation evaluation” were the main search terms used in the literature review. The China National Knowledge Infrastructure (CNKI) database, the Wanfang database, Web of Science, Engineering Index (EI), and other literature databases were used to retrieve the relevant literature. Duplicated articles were excluded, and the others were summarized in terms of their inclusion of a green performance evaluation index system, an index system of multi-agent cooperation evaluation, and different evaluation methods. Therefore, the literature was reviewed based on these three aspects.

### 2.1. Green Performance Evaluation Index System

As for the performance evaluation of green innovation, Arundel and Kemp (2009) constructed a green innovation performance evaluation index system from four aspects: Technology output, knowledge output, direct performance, and indirect performance [19]. Cheng and Shiu (2012) set up an evaluation system from the three dimensions of eco-organization innovation, eco-process innovation, and eco-product innovation [20]. Bi et al. (2013) constructed an evaluation index system of green process innovation performance from the three aspects of economic performance, social performance, and ecological performance [21]. The index system established by Tseng et al. (2013) includes management innovation, process innovation, product innovation, and technology innovation [22]. When measuring the efficiency of green innovation, Ghisetti and Rennings (2014) selected nine indicators, including energy consumption and environmental pollution [23]. Rumanti et al. (2017) built a new green innovation model containing knowledge sharing and open innovation by taking small- and medium-sized enterprises in Indonesia as case studies [24]. Guo et al. (2018) measured green technology innovation performance from the perspective of energy conservation and emission reduction [25]. In addition, Suresti et al. (2018) analyzed the maturity of the innovation system of the livestock industry in west Sumatra from three aspects: innovation policy, innovation procedures, and innovation capacity [26]. Sun et al. (2019) used the entropy weight Technique for Order Preference by Similarity to Ideal Solution (TOPSIS) method to measure China’s regional green innovation capacity [27]. The indicators established in these literature articles were used for reference to construct the indicators in this study. 

In terms of efficiency evaluation of green innovation, Lanoie et al. (2011) discussed the possibility that environmental efficiency could be affected by environmental policies [28]. Wong et al. (2012) discussed the impacts of green process innovation and green product innovation on green efficiency and the economic efficiency of green innovation, respectively [29]. Similarly, Wong (2013) analyzed the performance of green product innovation and green process innovation [30]. Ren and Wang (2016) used slack measure model (SBM) model to measure the green innovation efficiency of Chinese industrial enterprises [31]. Guo and Yang (2016) used the Super-Efficiency-SBM model and the three-stage DEA method to calculate the green innovation efficiency of different provinces in China [32]. Liu et al. (2016) used the range-adjusted measure (RAM)-DEA model to determine environmental efficiency in the coal industry [33]. Wang et al. (2017) pointed out that a lag of environmental efficiency is the main constraint factor for the green innovation performance of the manufacturing industry [34]. 

### 2.2. Index System of Multi-Agent Cooperation Evaluation

Many scholars have studied the function of multi-agent, the formation of collaboration, the action path, and the driving advantages. Seo and Hwang (2012) believed that strengthening the R&D intensity of multi-agent knowledge and technology cooperation is conducive to driving the development of vertical specialization [35]. Hewitt-Dundas (2013) believed that the dynamic feedback of technology application to knowledge innovation can increase the effectiveness of knowledge transfer from universities to enterprises [36]. Ruan et al. (2014) thought that regional clusters should promote knowledge exchange in high-tech enterprise cultivation [37]. As for the coordination mechanism, Jones and Zubielqui (2017) believed that university and research institutions should rely on the interaction and collaboration of industrial cooperative subjects [38]. Li et al. (2018) collected indicators for evaluating university and research institutions and industrial innovation [39]. Xu (2018) elaborated on the key links in the collaborative innovation process of university and research institutions and industrial innovation [40]. Tang et al. (2019) studied the direct relationship between university proximity and product innovation performance [41]. 

In terms of the formation, action path, and driving advantage of multi-agent collaboration, Salavisa et al. (2002) believed that the attributes of multi-agent collaboration include the difference of main body functions, resource distribution, resource allocation, and main body interaction [42]. Christian and Østergaard (2009) pointed out that multi-agent collaborative innovation is in accordance with the external environment and resources when making decisions [43]. Lewrick (2011) stated that the competitive orientation is highly correlated with progressive innovation [44]. Brettel et al. (2012) showed that both demand-responsive and demand-oriented factors have a positive effect on corporate performance [45]. As for the functional docking of homogeneous and heterogeneous subjects, Vick et al. (2015) pointed out that the innovation transformation process of knowledge and technology subjects has a network feedback relationship of subject externality [46]. Luo et al. (2015) showed that multi-agent collaborative innovation has the advantages of resource flow, integration network, and close innovation efficiency [47]. According to communication characteristics, Wu et al. (2017) highlighted that a specific investment indirectly acts on cooperative innovation performance through relationship trust [48]. The research results of Wang et al. (2018) showed that financial control is more effective for enterprises that specialize in cooperative innovation [49]. Wei and Chan (2019) believed that the cooperation stability of military and civilian organizations is positively correlated with government incentives [50]. 

### 2.3. Overview of Evaluation Methods

Many scholars have applied different evaluation methods to study the evaluation problems related to innovation and the manufacturing industry. Govindan et al. (2015) combined decision making trial and evaluation laboratory-analytic network process (DEMATEL-ANP) with the preferred ordering organization method to study production quality [51]. Lin et al. (2018) used the DEA window analysis method with ideal window width to evaluate the efficiency of green technology innovation [52]. Cao et al. (2018) evaluated the innovation performance of listed Integrated circuit companies by using the entropy weight method [53]. Han et al. (2018) studied the technology innovation performance of high-tech enterprises with the two-stage DEA model [54]. Nie et al. (2019) calculated the two-stage green innovation efficiency of Chinese industrial enterprises by using the DEA-SBM model [55]. Sun et al. (2019) measured China’s regional green innovation capacity by employing the entropy weight TOPSIS method [27]. 

In addition, there are many evaluation methods to determine manufacturing-related capabilities. Li et al. (2014) studied the development capability of China’s regional manufacturing industry by using the grey relational projection method with a combination of fuzzy-AHP–entropy weight [56]. Zhao et al. (2009) used the analytic hierarchy process to evaluate the sustainable development ability of the manufacturing industry [57]. Li et al. (2017) evaluated the green competitiveness of the manufacturing industry by employing the projection pursuit model of genetic algorithm [58]. Yu et al. (2015) evaluated the manufacturing quality competitiveness with the three-stage DEA model [59]. Cao et al. (2016) used collinearity and the coefficient of variation method to screen indicators, as well as the Delphi method to calculate the index weight [60]. Feng (2013) used the DEA-SBM method to measure green innovation efficiency [61]. Li et al. (2015) used the combined weighting method based on the FAHP and the maximum deviation method to calculate the index weight [62]. 

## 3. Construction of the Evaluation Index System

### 3.1. Characteristics of Green Technology Innovation Performance

A green technology innovation system of manufacturing enterprises under multi-agent cooperation is a complex system, which mainly includes manufacturing enterprises and multi-agent (universities, research institutions, government, intermediaries, upstream and downstream enterprises, consumers, etc.) [34,35,36,37,38,39,40]. Among them, manufacturing enterprises are the initiator and core subject of the system and determine the effect of the green technology innovation of manufacturing enterprises under multi-agent cooperation. The system is supported by many elements in order to function effectively. These elements include resource supply, capital supply, technical service, product demand, cooperation policy, and policy incentive [18,19,20,21]. At the same time, there are many factors and complex structures that affect the performance of the green technology innovation of manufacturing enterprises under multi-agent cooperation. Only by constructing an evaluation index system from multiple perspectives and levels can the green technology innovation performance of manufacturing enterprises under multi-agent cooperation be fully reflected.

**Figure 2:**
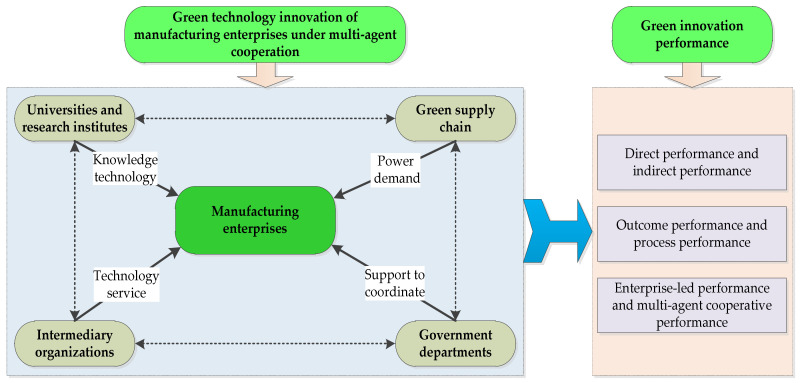
The performance formation framework of the green technology innovation performance of manufacturing enterprises under multi-agent cooperation.

From the perspective of formation, the complete green technology innovation performance should include two parts: The green technology innovation output performance and the green innovation process performance of manufacturing enterprises under multi-agent cooperation. From the multi-agent perspective, green technology innovation performance includes the leading performance of manufacturing enterprises and the multi-agent cooperation performance. When evaluating the green technology innovation performance of manufacturing enterprises under multi-agent cooperation, the direct and indirect performances brought about by multi-agent cooperation should be emphasized. The reciprocal cooperative relationship between manufacturing enterprises and multi-agent collaboration is an important index to evaluate the performance of the green technology innovation of manufacturing enterprises under multi-agent cooperation. Based on the above analysis, a framework of performance formation of manufacturing enterprises under multi-agent cooperation is shown in Figure 2. Figure 2 fully reflects the green technology innovation performance of manufacturing enterprises under multi-agent cooperation. The performance formation framework lays a theoretical foundation for the establishment of an evaluation index system of green technology innovation performance for manufacturing enterprises under multi-agent cooperation.

### 3.2. Design Principles for Evaluating Indicators

In order to accurately reflect performance, the index system should be constructed from multiple perspectives. The rationality and objectivity of the performance results should be guaranteed by following the principles outlined below.

(1) Scientific principle. The scientific principle means that the whole process of green technology innovation should be scientifically presented from multiple perspectives. In essence, it reflects the characteristics of the green technology innovation of manufacturing enterprises under multi-agent cooperation.

(2) Practicality principle. The practicability principle is shown in the following two aspects: Data collection and implemented evaluation. In the initial selection process of evaluation indicators, it is important to take into account the availability of data. In addition to ensuring the availability of data, it is also necessary to ensure the consistency of the statistical data caliber so that the evaluation can be operated and can be more practical.

(3) Combining the principles of comprehensiveness and conciseness. It is necessary to undertake comprehensive consideration when constructing an evaluation index system. The selection of indicators should cover all aspects of the green technology innovation of manufacturing enterprises under multi-agent cooperation. In addition, simple indicators with strong representativeness for evaluation should be chosen, and indicators with similar meanings or even duplications should be avoided.

(4) Comparability principle. In order to make sure the evaluation is representative, it is necessary to evaluate the performance of the green technology innovation of many manufacturing enterprises under multi-agent cooperation. This means that comparisons between manufacturing enterprises are essential. This principle should be fully taken into account in the construction of an evaluation index system.

(5) Propose principles for countermeasures. The purpose of green technology innovation performance evaluation is to find any issues in the process of the green technology innovation of manufacturing enterprises under multi-agent cooperation. According to the evaluation results, many countermeasures are put forward to improve said green technology performance. The selection of evaluation indicators should also be considered to help the government to develop green innovation policies.

(6) Principle of green development. Green development refers to the shift to cleaner and more efficient technologies, as close as possible to zero emissions or closed process methods, and to as low as possible consumption of energy and other natural resources. The performance evaluation of the green technology innovation of manufacturing enterprises under multi-agent cooperation should be considered in favor of the principle of enterprise green development.

### 3.3. Predictive Test Indicators

The aspects of green performance evaluation index system, index system of multi-agent cooperation evaluation, and the use of different evaluation methods were reviewed to lay a foundation for the construction of the index system. In addition, Tsai and Liao (2014) and Vukšić (2015) proposed an evaluation framework for open innovation [63,64]. In the research of Kobarg et al. (2018), objective indicators were used to measure the cooperation performance [65]. Szucs (2018) emphasized the use of subjective indicators that focus on evaluating the cognitive outcomes of participants [66]. Biedenbach et al. (2018) proposed a conceptual evaluation model of industry–university–research cooperation performance [67]. Witte et al. (2018) pointed to the importance of factors such as capital, collaboration, and proximity [68]. Li et al. (2018) constructed the input-output-transformation process model of collaborative innovation [69]. The above-mentioned literature emphasizes the importance of enterprise-led performance and multi-agent cooperation performance, most of which focus on the innovation input, the innovation output, the economic output of innovation, and the social effects of innovation. 

The connotation of performance is mainly embodied in the three aspects of effect, efficiency, and benefit. The effect is the degree to which the goal is achieved, which is the appearance of the performance; the efficiency is the relationship between input and output; and the benefit refers to the economic, social, and environmental benefits that the final result brings to the organization or individual [27,28,29,30,31,32,33]. However, scholars have not yet reached a consensus regarding the definition of innovation performance, which can be summarized from the following four aspects [62,63,64,65,66,67,68]. First, innovation performance is the increase of innovation output; second, it includes the whole process from the generation of new ideas to the introduction of inventions to the market; third, it is the efficiency of innovation input into output; fourth, it is determined by innovation output and innovation efficiency, both of which are equally important for innovation performance. Based on the above analysis, this study believes that innovation performance refers to the innovation achievements made by the innovation subject through a series of innovative activities, which can be reflected by technology output, economic output, and social effects.

The green technology produced by manufacturing enterprises and university and research institutions, as well as the application of customer-oriented technology, mainly reflect the green technology produced by green technology innovation. The economic output of the green technology innovation of manufacturing enterprises under multi-agent cooperation is a measure of the innovative benefits gained by these manufacturing enterprises through cooperative application of green technology practices with research institutions and active purchase of green products by consumers. These benefits also include government incentives and subsidies based on emissions reductions. The social effect of the green technology innovation of manufacturing enterprises under multi-agent cooperation include macroeconomic, green society, resource, and environmental effects brought by green technology innovation [23,24,25,26]. The macroeconomic effect is reflected in the radiation effect led by enterprises; the green society effect manifests itself in the public green environmental protection consumption consciousness and so on; the environmental effect is mainly reflected in the aspects of resource saving and waste reduction. Therefore, an evaluation index system of the green technology innovation performance of manufacturing enterprises under multi-agent cooperation can mainly be established from three perspectives: The technology output, the economic output, and the social effects of green technology innovation. The initial index system constructed is shown in Table 1.

There are many evaluation indicators in an initial evaluation index system, and the contribution of each index to green technology innovation and its own index quality are also different. Therefore, we constructed a comprehensive index system at the beginning of the study, and finally determined the formal indicators of this study through a series of tests and surveys. In Table 1, 32 indicators are summarized from the aspects of technology output, economic output, and social effects of green technology innovation. After determining the primary indicators, we adopted the expert scoring method, and divided the 32 indicators into very unimportant, relatively insignificant, unimportant, general, important, relatively important, and very important degrees according to the numerical order of 1 to 7. We asked four experts who have been studying the green technology innovation of manufacturing enterprises for a long time and six managers in charge of the green technology innovation projects of their manufacturing enterprise to jointly score the 32 indicators. After the first expert evaluation, the new evaluation form was returned to the experts for the second evaluation. After several rounds of comprehensive feedback, the final results tended to be consistent. We excluded the indicators with a mean score less than 5 through expert scoring. In the primary indicators, we excluded the sales revenue of green new products in the economic output of green technology innovation, and the customer satisfaction degree of green products in the social effect of green technology innovation. It should be noted that on a 7-point Likert scale, the number 1 is very unimportant and the number 7 is very important. The number of evaluation experts, different industries, and other factors may lead to some limitations of the evaluation results. Therefore, indicators greater than or equal to the value of 5 were selected first.

### 3.4. Formal Test Indicators

On the basis of indicator screening, we divided the questionnaire into two parts. The first part collected the basic information of the manufacturing enterprises in order to understand the basics such as company information (e.g., the industry, time of establishment, assets, level of profitability, etc.), company nature, and personnel composition. The second comprised the evaluation index system of the green technology innovation performance of manufacturing enterprises under multi-agent cooperation, focusing on the investigation of the green technology innovation projects under multi-agent cooperation led by manufacturing enterprises, thus requiring a comprehensive consideration of all green technology innovation projects. In order to obtain the research data, we chose technical and managerial personnel related to the green technology innovation projects as the research objects.

In order to ensure the validity and operability of the questionnaire, cities with certain social networks (such as Harbin, Daqing, Beijing, Shijiazhuang, Zhenjiang, etc.) in northeast China, north China, and the eastern coastal areas were selected as the research areas. We chose manufacturing enterprises with high demand for green technology innovation as the research objects. In order to ensure data quality from the source, the indicators in the relevant literature and the measurement indicators of major scholars were used to develop a pre-survey questionnaire. We distributed 50 questionnaires to MBA and EMBA students of Harbin Engineering University. The questionnaire was revised and adjusted accordingly. After the adjustment, a total of 400 questionnaires were issued, and 229 questionnaires were finally recovered—a recovery rate of 57.25%. After eliminating incomplete data and obviously wrong questionnaires, 188 valid questionnaires were finally obtained. The effective rate of the questionnaire was 82.10%, which basically met the requirements of index analysis. Technical personnel accounted for 33.25%; technical managers for 45.58%; middle and senior managers for 21.17%; and 84.14% of the total respondents had a bachelor’s degree or above.

In order to avoid any bias effects, homologous method bias and non-responder bias tests should be performed on the study data. The Harman single factor analysis method was used to test the deviation of the homologous method. The results of the exploratory factor analysis showed that the load of the first principal component without rotation was 17.96. There was no single factor that could explain most of the variation factors, and the homologous method bias did not have a significant effect. In terms of the non-responders’ deviation, the first 1/3 and the last 1/3 of the samples were selected for the *t*-test according to the return time of the questionnaire. The test results showed that there was no significant difference between more than 90% of the observed variables; thus, the deviation of non-responders did not have a significant impact. The Harman single factor analysis test and the *t*-test were implemented by using SPSS20.0 soft.

(1) Correlation analysis among indicators. After establishing an evaluation index system, it is necessary to investigate the correlation among these indicators. If two indicators are correlated, then these two indicators represent the same or similar content. Thus, one of the indicators should be eliminated to simplify the index system. Correlation analysis among indicators can be implemented by using SPSS20.0 soft and MATLAB soft. The correlation test results are shown in Table 2, Table 3 and Table 4.

1) As for the correlation analysis of the technology output index of green technology innovation, the correlation between the level of new green products and the proportion of national and provincial brand green products is 0.713, higher than 0.7. The number/level of new green products is a comprehensive reflection of green products, while the number of national and provincial famous green products is only a part of the comprehensive response. Therefore, only the number/level of new green products was selected.
2) As for the correlation analysis of the economic output index of green technology innovation, among the 10 indicators excluded, the growth rate index of the sales revenue of green products is correlated with the index of return on investment of green innovation projects; the correlation is 0.722, higher than 0.7. The return on investment indicators for green innovation projects can be used to reflect profit and revenue indicators. Therefore, the green product sales revenue growth rate index was excluded from the economic output index of green technology innovation.
3) As for the correlation analysis of the social effect index of green technology innovation, the social effect index of green technology innovation reflects the effect of green technology innovation from different aspects. There is no high correlation among the indicators.

(2) Exploratory factor analysis of a single index. In order to avoid the influence of excessive random error on the quality of indicators, it is necessary to evaluate the quality of said indicators. Generally speaking, reliability and validity tests are used to evaluate the rationality and reliability of index selection. Therefore, Cronbach’s alpha coefficient was used to test the reliability and validity of our indicators in order to ensure the measurement quality, the results for which were obtained by using SPSS20.0 soft.

Reliability $R=S_{t}^{2}/S_{r}^{2}$ is defined as the ratio of the true variance to the total variance of a test score, where R represents the reliability of the measurement. At the same time, since $S_{r}^{2}=S_{t}^{2}+S_{e}^{2}$, the reliability can also be expressed as $R=\left(S_{r}^{2}-S_{e}^{2}\right)/S_{r}^{2}=1-S_{e}^{2}/S_{r}^{2}$. Cronbach’s alpha $\alpha =\left[K/\left(K-1\right)\right]\left[1-\left(\sum S_{i}^{2}\right)/S_{r}^{2}\right]$ is an important measure of reliability. On the basis of correlation analysis, SPSS20.0 software was used for exploratory factor analysis. The results of the software analysis show that the Cronbach probability values of the technology output, the economic output, and the social effects of green technology innovation are 0.918, 0.896, and 0.870, respectively. The estimates of Cronbach of all kinds are greater than 0.75, indicating that the classification of the evaluation index system of the green technology innovation performance of manufacturing enterprises under multi-agent cooperation is reasonable. Moreover, most of the factor loads are higher than 0.7, and the variate interpretation variance is more than 70%. This suggests that the indicators can comprehensively reflect all aspects of green technology innovation performance.

A validity test is used to analyze the validity of a questionnaire’s measurement results. The purpose of this test is to determine whether the measurement results of the questionnaire reflect the objective reality it should reflect. To be specific, the validity test must be based on the specific purpose of the function and scope of application, as well as the different aspects of the collection of information. The commonly used validity tests include content validity, structure validity, difficulty validity, and criterion validity. We should judge the content validity, structure validity, and criterion validity of the questionnaire, which can be combined into a single concept of construct validity. Construct is a concept constructed by researchers according to research needs. Construct validity verifies the measurement of a concept by examining the measurement results of the concept. The scale has good construct validity if the score of the subject can effectively explain its psychological characteristics. In the process of constructing the scale, we established the construct validity of the index system. Therefore, this questionnaire is valid.

(3) Formal evaluation index system. Correlation analysis and exploratory factor analysis were conducted in order to obtain the final evaluation index system of the green technology innovation performance of manufacturing enterprises under multi-agent cooperation, as shown in Table 5. In the final system, the technology input of green technology innovation contains eight indicators, the economic output of green technology innovation contains nine indicators, and the social effects of green technology innovation contains nine indicators. Each dimension contains both quantitative and qualitative indicators. These three aspects not only reflect the balance between short-term and long-term goals, but also the effective combination between financial and non-financial indicators. The final system helps to fully reflect the direct performance and indirect performance, innovation output performance and innovation process performance, and enterprise-led performance and multi-agent cooperation performance. The formal evaluation index system in this study was used to truly evaluate the performance of the green technology innovation of manufacturing enterprises under multi-agent cooperation.

## 4. Methodology

### 4.1. Determination of the Evaluation Method

The existing evaluation methods are single evaluation methods. In this study, a secondary combined evaluation model was constructed based on the evaluation conclusions to evaluate the performance of the green technology innovation of manufacturing enterprises under multi-agent cooperation. First, the single evaluation methods of entropy weight, TOPSIS, and deviation maximization were used in this study. On this basis, the combined evaluation methods—i.e., a combination of the drift degree, grey relational degree, and mean value methods—were constructed to evaluate the performance of green technology innovation. Furthermore, the mean variance method and the correlation coefficient of Spearman’s grade were used to test the convergence of the combined evaluation results. If the consistency was strong, the secondary combined evaluation method was chosen according to the least deviation degree method. At the same time, secondary combined evaluation was carried out. The convergence test of quadratic combination results was carried out by the method of whether the mean value of variance is attenuated or not. The secondary combined evaluation model based on the evaluation conclusion can effectively improve the consistency and convergence of the first combined evaluation conclusion. At the same time, it can also not only reduce the combined error of the primary combined evaluation, but also improve the reliability of the overall evaluation conclusion.

### 4.2. Secondary Combined Evaluation Model

#### 4.2.1. Construction Idea of the Evaluation Model

**Step 1:** The original data of the evaluation indicators of the green technology innovation performance of manufacturing enterprises under multi-agent cooperation are standardized.

**Step 2:** The entropy weight method, the TOPSIS method, and the maximum deviation method are used for single evaluation. Spearman’s rank correlation coefficient is selected to test the consistency of the evaluation results. If the consistency is strong, the drift degree combined evaluation method, the grey relational degree combined evaluation method, and the mean value combined evaluation method are used to conduct the first combined evaluation.

**Step 3:** The consistency test based on Spearman’s rank correlation coefficient is carried out for the first combined evaluation results. If the consistency is strong, the secondary combined evaluation method is chosen according to the least deviation degree method. At the same time, the secondary combined evaluation is carried out.

**Step 4:** The convergence test of the quadratic combination results is carried out by the method of whether the mean value of variance is attenuated or not.

**Figure 3:**
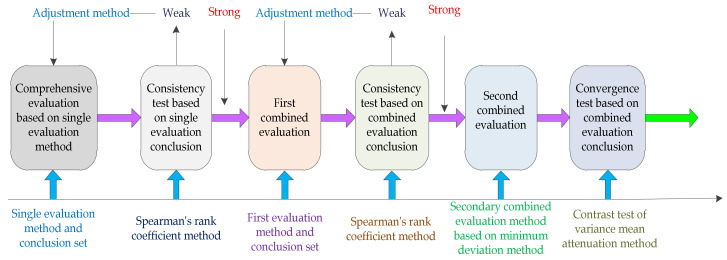
The construction idea of the secondary combined evaluation model.

The specific steps are shown in Figure 3.

#### 4.2.2. Index Normalization

$v_{ij}$ is the index j of the system i. $\alpha_{ij}$ represents the upper limit value of the system critical point order parameter, and $\beta_{ij}$ represents the lower limit value of the system critical point order parameter. $p_{ij}$ is the contribution of the variable $v_{ij}$ to the system, and $p_{ij}\in [0,1]$. Therefore, the efficacy coefficients of positive and negative indicators are as follows”

(1) $$p_{ij}=\left\{\begin{array}{l} \left(v_{ij}-\beta_{ij})/(\alpha_{ij}-\beta_{ij}\right)\\ \left(\alpha_{ij}-v_{ij})/(\alpha_{ij}-\beta_{ij}\right) \end{array}\right.$$

#### 4.2.3. Single Evaluation Methods

(1) The entropy weight method. The specific steps of the entropy weight method are as follows:

1) Set $f_{ij}$ as the index specific gravity, and the calculation formula is as follows:

(2) $$f_{ij}=v_{ij}/\sum_{i=1}^{n}v_{ij}$$

where $v_{ij}$ is the original data of the evaluation index, and i=1,2,…,n; j=1,2,…,m.

2) If $h_{j}$ is set as the entropy value of the evaluation index j and $w_{j}$ is the weight of the evaluation index j, the relationship is as follows:

(3) $$w_{j}=\left(1-h_{j}\right)/\left(m-\sum_{j=1}^{m}h_{j}\right)$$

where $h_{j}=-\frac{1}{\ln n}\sum_{i=1}^{n}f_{ij}\ln(f_{ij})$.

3) Suppose $P_{i}$ represents the performance score of green technology innovation, then the score calculation formula is as follows:

(4) $$P_{i}=\sum_{j=1}^{m}w_{j}v_{ij}$$

(2) The TOPSIS method. The specific steps of the TOPSIS method are as follows:

1) Construct the index weighting matrix. If the set of manufacturing enterprises is M and the included set of indicators is S, then the value of $M_{i}$ to $S_{j}$ is denoted as $p_{ij}$. The weighted index weights $w_{j}$ and the dimensionless matrix are multiplied to obtain the weighted matrix $R={\left(r_{ij}\right)}_{m\times n}$, where $r_{ij}=w_{j}\times p_{ij}$.

2) Calculate the positive and negative ideals of the green technology innovation performance. Based on the weighted matrix, the positive and negative ideal values are calculated as follows:

(5) $$Y_{j}^{+}=\underset{1\leq i\leq m}{\max}\left\{r_{ij}\right\},Y_{j}^{-}=\underset{1\leq i\leq m}{\min}\left\{r_{ij}\right\}$$

Thus, the positive ideal solution $Y^{+}=(Y_{1}^{+},Y_{2}^{+},\ldots ,Y_{m}^{+})$ and the negative ideal solution $Y^{-}=(Y_{1}^{-},Y_{2}^{-},\ldots ,Y_{m}^{-})$ for the green technology innovation performance can be obtained.

3) Calculate the Euclidean distance. If $d_{i}^{+}$ is the positive ideal score and $d_{i}^{-}$ is the negative ideal score, the relationship is as follows:

(6) $$d_{i}^{+}=\sqrt{{\left(Y_{1}^{+}-Y_{1i}\right)}^{2}+\ldots +{\left(Y_{m}^{+}-Y_{mi}\right)}^{2}},d_{i}^{-}=\sqrt{{\left(Y_{1}^{-}-Y_{1i}\right)}^{2}+\ldots +{\left(Y_{m}^{-}-Y_{mi}\right)}^{2}}$$

4) The performance score of green technology innovation. Calculate the relative closeness $C_{i}=d_{i}^{-}/(d_{i}^{+}+d_{i}^{-})$. The higher the value of $C_{i}$, the higher the green technology innovation performance.

(3) The maximum deviation method. The steps of maximum deviation method are as follows:

1) Let $v_{ij}(i=1,2,\ldots ,n;j=1,2,\ldots ,m)$ represent the value of the index j of the evaluation object i. Let $w_{j}$ be the weight of the index j, $w_{j}\geq 0$. For index j, $E_{ij}\left(w\right)$ represents the total deviation (k=1,2,…,n) between object i and all other object index values. Then, the calculation formula is as follows:

(7) $$E_{ij}\left(w\right)=\sum_{j=1}^{m}\left|v_{ij}w_{j}-v_{ik}w_{j}\right|$$

2) Let $E_{j}\left(w\right)$ represent index j, and the total deviation between the objects and all other objects is as follows:

(8) $$E_{j}\left(w\right)=\sum_{i=1}^{n}E_{ij}\left(w\right)=\sum_{i=1}^{n}\sum_{k=1}^{n}\left|vi_{ij}-v_{ik}\right|w_{j}$$

3) Under the condition of maximum total deviation, the objective function of the weighted vector w is as follows:

(9) $$\begin{array}{l} maxE\left(w\right)=\sum_{j=1}^{m}\sum_{i=1}^{n}\sum_{k=1}^{n}\left|v_{ij}-v_{ik}\right|w_{j}\\ s.t.\left\{\begin{array}{l} w_{j}\geq 0\\ \sum_{j=1}^{m}w_{j}{}^{2}=1 \end{array}\right. \end{array}$$

4) Apply the Lagrange function to the optimization model and obtain its partial derivative. The normalized weight vector is obtained as follows:

(10) $$w_{j}=\frac{\sum_{i=1}^{n}\sum_{k=1}^{n}\left|v_{ij}-v_{ik}\right|}{\sum_{j=1}^{m}\sum_{i=1}^{n}\sum_{k=1}^{n}\left|v_{ij}-v_{ik}\right|}$$

5) Let $V_{i}$ be the comprehensive score of each evaluation object, and the calculation formula is as follows:

(11) $$V_{i}=\sum_{j=1}^{m}w_{j}v_{ij}$$

#### 4.2.4. Combined Evaluation Methods

(1) The drift degree method. The steps of drift degree method are as follows.

1) The average value of each single evaluation method is used as the reference frame for the drift measurement. The correlation coefficient $r_{j}\left(t_{k}\right)$ of the evaluation value of each single evaluation method $u_{ij}\left(t_{k}\right)$ and the reference frame $\bar{u}\left(t_{k}\right)$ at time $t_{k}$ can be obtained. The drift degree of each single evaluation method is as follows:

(12) $$p_{j}\left(t_{k}\right)=1-r_{j}\left(t_{k}\right),j=1,2,\ldots ,b$$

2) According to the evaluation method, if it has large drift, it is given a small weight; otherwise, the opposite principle is applied. Then, the weight of method j at time $t_{k}$ is as follows:

(13) $$w_{j}\left(t_{k}\right)=\frac{\underset{1\leq j\leq b}{\min}\left[p_{j}\left(t_{k}\right)\right]+\underset{1\leq j\leq b}{\max}\left[p_{j}\left(t_{k}\right)\right]-p_{j}\left(t_{k}\right)}{\sum_{j=1}^{b}\left\{\underset{1\leq j\leq b}{\min}\left[p_{j}\left(t_{k}\right)\right]+\underset{1\leq j\leq b}{\max}\left[p_{j}\left(t_{k}\right)\right]-p_{j}\left(t_{k}\right)\right\}}$$

where j=1,2,…,b;k=1,2,…,N.

3) Assuming the evaluation conclusion $u_{ij}\left(t_{k}\right)$ is to be evaluated at time $t_{k}$, the combined evaluation result based on the weight $w_{j}\left(t_{k}\right)$ of the drift degree is as follows:

(14) $$P_{i}\left(t_{k}\right)=\sum_{j=1}^{b}u_{ij}\left(t_{k}\right)w_{j}\left(t_{k}\right),i=1,2,\ldots ,m$$

(2) The grey relational degree method. The method is mainly divided into the following steps:

1) The evaluation result of the single evaluation method is $x_{1},x_{2},\ldots ,x_{m}$, where i=1,2,…,m. Let $x_{0}$ be the ideal evaluation result, then the correlation coefficient between $x_{0}$ and $x_{i}$ about the k element is as follows:

(15) $$\xi_{i}\left(k\right)=\frac{\Delta \min+\rho \Delta \max}{\Delta_{i}\left(k\right)+\rho \Delta \max}，i=1,2,\ldots ,n;k=1,2,\ldots ,m$$

where $\Delta \min=\underset{i}{\min}\left\{\underset{k}{\min}\left[\left|x_{0}\left(k\right)-x_{i}\left(k\right)\right|\right]\right\}$ and $\Delta \max=\underset{i}{\max}\left\{\underset{k}{\max}\left[\left|x_{0}\left(k\right)-x_{i}\left(k\right)\right|\right]\right\}$. ρ is 0.5.

2) The correlation between i and the ideal evaluation results is as follows:

(16) γi=∑k=1mwξkik

where $w_{k}$ is the weight of i single evaluation result.

(3) The mean value method. The evaluation result of the green technology innovation performance of manufacturing enterprises is as follows:

(17) $$\bar{X}=\sum_{j=1}^{m}x_{ij}/n$$

#### 4.2.5. Determination and Test of the Secondary Combined Evaluation Method

(1) The determination of the secondary combined evaluation method

The sum of the squared error between the combined evaluation value of all evaluation objects $S_{i}$ and $\bar{r_{i}}$ is as follows:

(18) $$SSM_{tk}=\sum_{i=1}^{n}{\left(\bar{r_{i}}-r_{it}\right)}^{2}\;i=1,2,\ldots ,m;t=1,2,\ldots ,r;k=1,2,\ldots ,r$$

If the deviation degree of the evaluation result of a certain combination is the smallest, the method is relatively optimal.

(2) The test of the secondary combined evaluation method

1) The convergence test. The formulas for calculating the mean variance of the performance ranking results under the primary and secondary combined evaluation are as follows:

(19) $$S_{mean\times 1}^{2}=\frac{1}{m}\sum_{i=1}^{m}s_{i}^{2};S_{mean\times 2}^{2}=\frac{1}{m}\sum_{i=1}^{m}s_{j}^{2}$$

If $S_{mean\times 2}^{2}<S_{mean\times 1}^{2}$, the result of the secondary combined evaluation is considered to be better than that of the first.

2) The Spearman’s test. The correlation coefficient of Spearman’s level is used to check the consistency, and the formula is as follows:

(20) $$r_{ij}=1-\left[6\sum_{s=1}^{n}d_{s}^{2}/n\left(n^{2}-1\right)\right]$$

If $r_{ij}>0$, it indicates that there is a positive correlation between the two methods. If $r_{ij}<0$, it indicates that there is a negative correlation. If $r_{ij}=0$, the two methods are not related.

## 5. Results and Discussion

**Figure 4:**
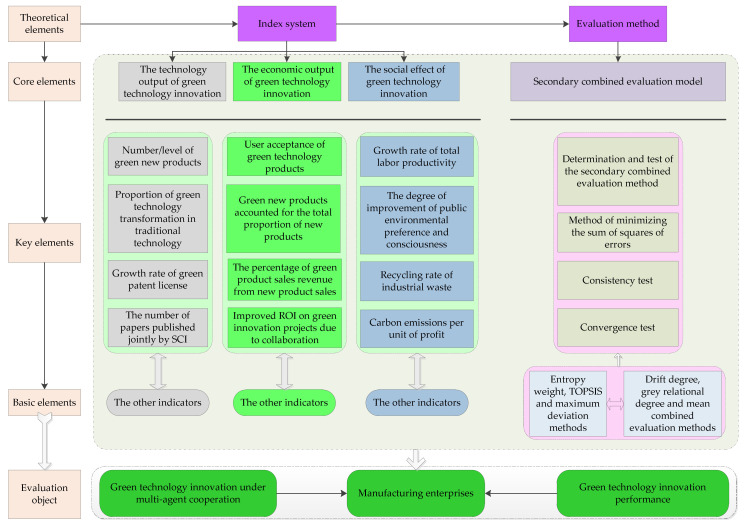
The theoretical framework to measure the performance of the green technology innovation of manufacturing enterprises under multi-agent cooperation.

The purpose of this study was to determine how to improve the effectiveness of multi-agent cooperation in China. We proposed a theoretical framework to measure the performance of green technology innovation, which includes an evaluation system and an evaluation method of the green technology innovation performance of manufacturing enterprises under multi-agent cooperation, and is represented in Figure 4. 

### 5.1. Analysis of the Evaluation Index System

As shown in Figure 4, the evaluation system of the green technology innovation performance of manufacturing enterprises under multi-agent cooperation includes core elements, key elements, and basic elements. The core elements represent the green innovation performance; the key elements are the key factors that affect the performance of green innovation; the basic elements are an indispensable part of green innovation performance.

#### 5.1.1. Analysis of the Core Elements

As shown in Figure 4, the evaluation system of the green technology innovation performance of manufacturing enterprises under multi-agent cooperation is composed of the technology output, the economic output, and the social effects of green technology innovation. These three aspects correspond to the innovation value chain proposed by Hansen and Birkinshaw [73]. The innovation value chain is an activity flow from the generation of ideas to commodities, which helps to improve the performance of multi-agent cooperation. Therefore, the green technology innovation process of manufacturing enterprises under multi-agent cooperation is divided into three stages: The technology output stage, the economic output stage, and the social effects stage. The technology output stage of green technology innovation corresponds to the generation of ideas and the basic research of green technology application; the economic output stage of green technology innovation corresponds to the transformation of ideas and the development of green technology; the social effects stage of green technology innovation corresponds to the dissemination of ideas and the environmental benefits of green technology products.

Specifically, the technology output of green technology innovation includes the cooperation between manufacturing enterprises, universities, and research institutions to carry out basic research activities on the application of green technology. The economic output of green technology innovation includes the cooperation between manufacturing enterprises, universities, research institutions, and technology intermediaries to carry out green technology R&D activities, such as knowledge technology development and technology productization. The social effects of green technology innovation mainly refer to the targeted and effective work arrangement between manufacturing enterprises, downstream enterprises, and consumers in terms of product promotion and industrial development to achieve environmental benefits. Multi-stage innovation input and feedback make the whole innovation process and the final production system form a kind of dynamic evolutionary competitive advantage. The competitive advantage is the green technology innovation performance of manufacturing enterprises under multi-agent cooperation.

#### 5.1.2. Analysis of the Key Elements

As shown in Figure 4, the key factors that influence the technology output of green technology innovation include the number/level of new green products, the proportion of green technology transformation in traditional technology, the number of papers published jointly by multi-agent cooperation, and the green technology achievement conversion rate. As shown in Table 5, the proportion of green technology transformation in traditional technology and the number of papers published jointly by multi-agent cooperation are the most important factors. These two factors indicate that China’s traditional manufacturing industry is developing gradually in terms of green transformation and green innovation with the process of technology advancement. Innovation is one of the most important decisive factors in the comprehensive implementation of green manufacturing. At present, the transformation of the traditional manufacturing industry is the best way to develop China’s manufacturing industry. Instead of simply increasing the quantity and expanding the scale to achieve this development, China has begun to vigorously implement green technology innovation and promote the upgrading and greening of manufacturing capacity. At the same time, China has introduced a number of policies to emphasize the important role of strengthening green technology innovation and accelerating the R&D of applied technologies. Indeed, many manufacturing enterprises are actively implementing integrated and systematic green solutions that can coordinate energy conservation, consumption reduction, emission reduction, and pollution control.

The key factors that influence the economic output of green technology innovation include the new green products accounting for the total proportion of new products, the percentage of green product sales revenue from new product sales, the user acceptance of green technology products, and the improved ROI on green innovation projects due to collaboration. As shown in Table 5, the user acceptance of green technology products is the most important among these factors. This factor indicates that consumers in China are not willing to buy green products. In fact, China has introduced many policies to promote the transformation of green technology into real productivity. On the one hand, manufacturing enterprises are given tax incentives, government subsidies, and other measures to promote green technology innovation and product sales. On the other hand, green consumers are given purchase subsidies, convenient travel, and other measures to promote their purchase of green products. For example, the Chinese government has subsidized the purchase of new energy vehicles since 2009. The Chinese government studies the incentive and subsidy policies to optimize the construction of charging facilities to create a favorable environment for the consumption of new energy vehicles.

The key factors that influence the social effects of green technological innovation include the growth rate of total labor productivity, the degree of improvement of public environmental preference and consciousness, and the recycling rate of industrial waste, and the carbon emissions per unit of profit. As shown in Table 5, the degree of improvement of public environmental preference and consciousness is the most important among these factors. This factor indicates that public participation is an important factor affecting green innovation under multi-agent cooperation. Public awareness of environmental protection plays a decisive role in realizing the social effects of green technology innovation. Only with the active participation and joint efforts of the public can the development of green effects be truly realized. This needs not only the continuous enhancement of the public awareness of environmental protection, but also to enable the broad masses of the people to consciously improve their consumption concept and behavior.

### 5.2. Analysis of the Evaluation Method

As shown in Figure 4, the evaluation method of the green technology innovation performance of manufacturing enterprises under multi-agent cooperation includes core elements, key elements, and basic elements. Core elements refer to the core ideas of the secondary combined evaluation model; key elements are the basic principles of the secondary combined evaluation model; basic elements are the necessary parts in the secondary combined evaluation model. The secondary combined evaluation model proposed in this study was used to evaluate the green technology innovation performance of manufacturing enterprises under multi-agent cooperation. 

#### 5.2.1. Analysis of the Method Characteristics

The purpose of combined evaluation is to combine the evaluation conclusions obtained by multiple single evaluation methods to reduce the random error and systematic deviation. This kind of evaluation can make best use of the information of multi-evaluation conclusions to improve the accuracy and credibility of evaluation conclusions. The basic idea is to combine the evaluation results of several representative evaluation methods with appropriate ideas or methods. The results of combined evaluation are obtained according to the value of the combined evaluation. The combination of evaluation values is more direct than the combination of evaluation weights, and the deviation caused by the results of weight combination can be avoided. Compared with the ordinal value combination, the combination of evaluation values takes into account more information, which makes the evaluation value of the combination closer to the real value.

The secondary combined evaluation is a type of combined evaluation based on the evaluation conclusions and combines the evaluation results obtained by each single evaluation method in a certain form. The conclusion of the secondary combined evaluation is close to the real value, which improves the authenticity and reliability of the evaluation results. The secondary combined evaluation is under the systematic framework of the combined evaluation and is based on the consistency of the results of the first combined evaluation. The relatively optimal combined evaluation method is used to recombine the results of the first combined evaluation, with the purpose of obtaining the evaluation value of the comprehensive evaluation problem of multiple indicators and multiple schemes.

#### 5.2.2. Analysis of the Method Validity

Based on the idea of combined evaluation, the concept of secondary combined evaluation is put forward. The aim of secondary combined evaluation is to choose the best combined evaluation method to recombine the results of the first combined evaluation. The purpose of this method is to make full use of suboptimal information and to improve the consistency and convergence of the first evaluation. At the same time, the selection rule of the secondary combined evaluation method is established. This study uses the simple arithmetic mean value of all evaluation values as a benchmark to represent the real value of the evaluation object according to the statistical theory. The concept of the deviation degree is put forward to measure the deviation degree between the measured value of the combined evaluation method and the real value of the evaluated object. Then, the error squared sum is used to measure the deviation degree according to the error theory.

In order to verify the validity of the proposed secondary combined evaluation model, we carried out a comparative analysis. The data of the effective technical supply level in China were used to verify the validity. The exact process is shown in Annex 1. Before the secondary combination, it was necessary to determine a reasonable evaluation method of secondary combination. Equation (18) was used to calculate the deviation degree, and the results were $SSM_{11}=0.1960$, $SSM_{12}=0.1947$ and $SSM_{13}=0.7803$, respectively. Obviously, the combined error sum of the mean combined evaluation method is the smallest. Therefore, this method was selected for secondary combined evaluation, and the results are shown in Table 6.

In order to compare the combination effect of the first combined evaluation results with the secondary results, the squared sum $SSM_{t}$ of deviations was used to test the deviation. We assumed that the average of the results of the first and secondary combined evaluations is the real value. The error sum of the drift degree combined evaluation method, the mean value combined evaluation method, and the grey relational degree combined evaluation method are, respectively, $SSM_{21}=\begin{array}{l} 0.196042 \end{array}$, $\begin{array}{l} SSM_{22}= \end{array}\begin{array}{l} 0.194728 \end{array}$, $SSM_{23}=0.780250$, and $SSM_{3}=0.000000$—namely, $SSM_{3}<SSM_{22}<SSM_{21}<SSM_{23}$. It can be seen that the error sum of the secondary combination is the smallest and the combination effect is better than the first. In terms of convergence, the average variance of the combined evaluation methods is $S_{mean\times 1}^{2}=0.1000>S_{mean\times 2}^{2}=0.0778$. The results show that the evaluation results of the secondary combination tend to converge, which are better than those of the first combined evaluation. The two results show that the secondary combined evaluation method can not only effectively improve the consistency and convergence but can also improve the reliability of the overall evaluation conclusion.

## 6. Conclusions and Future Research Directions

On the basis of “Made in China 2025”, the Chinese government has further formulated a series of documents, including the Industrial Green Development Plan (2016–2020) and the Green Manufacturing Project Implementation Guide (2016–2020), to further promote the development of green manufacturing. How should this process reflect more systematically and objectively the quality of the manufacturing economy driven by green innovation? Is it necessary to establish an evaluation index system of green technology innovation performance to match the requirements of ecological civilization construction? The solution to this kind of problem becomes the key point to perfect the green innovation mechanism of the manufacturing industry. Therefore, this study constructed a theoretical framework to measure the performance of the green technology innovation of manufacturing enterprises under multi-agent cooperation. The theoretical framework includes an evaluation system and an evaluation method of green technology innovation performance. The evaluation index system of the green technology innovation performance of manufacturing enterprises under multi-agent cooperation includes the technology output, the economic output, and the social effects of green technology innovation. The secondary combined evaluation model includes single evaluation methods and combined evaluation methods. The secondary combined evaluation method was chosen according to least deviation degree method to measure the performance of the green technology innovation of manufacturing enterprises under multi-agent cooperation.

The results of this study are as follows. The green technology innovation process of manufacturing enterprises under multi-agent cooperation includes three stages: the technology output stage, the economic output stage, and the social effects stage. The evaluation index system of the green technology innovation performance of manufacturing enterprises under multi-agent cooperation is composed of the technology output, the economic output, and the social effects of green technology innovation. The key factors that influence the technology output include the number/level of new green products, the proportion of green technology transformation in traditional technology, the number of papers published jointly by multi-agent cooperation, and the green technology achievement conversion rate. The key factors that influence the economic output include the new green products accounting for the total proportion of new products, the percentage of green product sales revenue from new product sales, the user acceptance of green technology products, and the improved ROI on green innovation projects due to collaboration. The key factors that influence the social effects include the growth rate of total labor productivity, the degree of improvement of public environmental preference and consciousness, the recycling rate of industrial waste, and the carbon emissions per unit of profit. A fusion of technology of subjective and objective methods can be adopted to evaluate the performance of green technology innovation. The secondary combined evaluation is a combined evaluation based on the evaluation conclusions, which combines the evaluation results obtained by each single evaluation method. The concept of the deviation degree is put forward to measure the deviation degree between the measured value of the combined evaluation method and the real value.

This study has important theoretical and practical significance. The systematic construction of an evaluation index system and the method of green technology innovation performance not only fully consider the characteristics of multi-agent cooperation, but also expand the theoretical system and the perspective of existing innovation performance evaluation research. Manufacturing enterprises under multi-agent cooperation can not only effectively carry out activities of green knowledge creation, green technology transformation, and green product promotion, but can also continuously improve the green value creation effect of multi-agent cooperation led by manufacturing enterprises. In addition, this study can help the government to formulate reasonable policies to support the development of the green technology innovation of manufacturing enterprises under multi-agent cooperation.

Although this study has established an evaluation index system and an evaluation model, there are still some shortcomings. Due to the lack of data, it is impossible to conduct extensive and sufficient practical tests in the process. In the future, empirical research on the green innovation of manufacturing enterprises under multi-agent cooperation should be carried out, and the proposed system and model should be fully tested and improved.

## Figures and Tables

**Table 1:** The initial evaluation index system of the green technology innovation performance of manufacturing enterprises under multi-agent cooperation.

Dimension	Serial Number	Index Layer	Cooperation Subjects and Main References
The technology output of green technology innovation	1/2	Number/level of new green products	Cooperation subjects: Universities, scientific research institutions, intermediary agencies, green suppliers, green consumers, etc.Main references: [19], [26], [58], [70]
	3/4	Number of green invention patent applications/levels	
	5	Proportion of green technology transformation in traditional technology	
	6	Growth rate of number of green patent licenses	
	7	Proportion of green projects in the number of new product development projects	
	8	Number of papers published jointly by multi-agent cooperation	
	9	Number of joint application projects	
	10	Number of green products of national and provincial famous brands accounting for the proportion	
	11	Green technology achievement conversion rate	
The economic output of green technology innovation	12	New green products accounting for the total proportion of new products	Cooperation subjects: Supply chain enterprises, green consumers, government departments, etc.Main references: [19], [21], [25], [70,71]
	13	New green product sales revenue	
	14	Export of new green products creating exchange rate	
	15	Percentage of green product sales revenue from new product sales	
	16	Market share of new green products	
	17	Government incentives and subsidies based on emission reductions	
	18	User acceptance of green technology products	
	19	Improved return on investment (ROI) on green innovation projects due to collaboration	
	20	Improvement in the success rate of new green product development through cooperation	
	21	Increased net profit margin due to cooperation	
	22	Growth rate of green product sales	
The social effect of green technology innovation	23	Reduction rate of resource consumption per unit profit	Cooperation subjects: A series of green innovation activities with multi-agent participationMain references: [21], [23], [59,60], [72]
	24	Rate of reduction in energy consumption per unit of profit	
	25	Green product customer satisfaction	
	26	Growth rate of total labor productivity	
	27	Degree of improvement in public environmental preference and consciousness	
	28	Adoption of environmental management systems	
	29	Recycling rate of industrial waste	
	30	Carbon emissions per unit of profit	
	31	Discharge of three waste pollutants per unit profit	
	32	Forming national or industrial green technology standards	

**Table 2:** The relevance test of the technology output indicators of green technology innovation.

	X1	X2	X3	X4	X5	X6	X7	X8	X9	X10	X11
X1	1										
X2	0.581	1									
X3	0.487	0.490	1								
X4	0.595	0.594	0.479	1							
X5	0.608	0.612	0.466	0.625	1						
X6	0.589	0.592	0.523	0.489	0.578	1					
X7	0.396	0.497	0.494	0.504	0.551	0.478	1				
X8	0.626	0.606	0.484	0.611	0.624	0.600	0.492	1			
X9	0.574	0.507	0.402	0.524	0.539	0.519	0.405	0.553	1		
X10	0.650	0.713	0.457	0.630	0.593	0.530	0.454	0.557	0.538	1	
X11	0.518	0.578	0.452	0.569	0.571	0.544	0.422	0.553	0.475	0.572	1

**Table 3:** The relevance test of the economic output indicators of green technology innovation.

	X12	X13	X14	X15	X16	X17	X18	X19	X20	X21	X22
X12	1										
X13	0.425	-									
X14	0.491	-	1								
X15	0.515	-	0.515	1							
X16	0.475	-	0.384	0.437	1						
X17	0.478	-	0.441	0.493	0.538	1					
X18	0.638	-	0.480	0.549	0.619	0.533	1				
X19	0.506	-	0.462	0.604	0.449	0.520	0.473	1			
X20	0.556	-	0.340	0.471	0.569	0.485	0.538	0.562	1		
X21	0.428	-	0.403	0.518	0.373	0.413	0.471	0.502	0.402	1	
X22	0.568	-	0.432	0.609	0.503	0.497	0.522	0.722	0.573	0.472	1

**Table 4:** The relevance test of the social effect indicators of green technology innovation.

	X23	X24	X25	X26	X27	X28	X29	X30	X31	X32
X23	1									
X24	0.512	1								
X25	-	-	-							
X26	0.424	0.402	-	1						
X27	0.448	0.428	-	0.627	1					
X28	0.405	0.328	-	0.483	0.491	1				
X29	0.427	0.259	-	0.354	0.433	0.283	1			
X30	0.475	0.483	-	0.567	0.616	0.413	0.369	1		
X31	0.323	0.342	-	0.472	0.465	0.346	0.207	0.455	1	
X32	0.391	0.374	-	0.559	0.516	0.433	0.360	0.553	0.486	1

**Table 5:** The formal evaluation index system of the green technology innovation performance of manufacturing enterprises under multi-agent cooperation.

Dimension	Serial Number	Index Layer	Factor Loading	Variance of Interpretation (%)	Internal Consistency (α)
The technology output of green technology innovation	1/2	Number/level of new green products	0.79	76.51	0.918
	3/4	Number of green invention patent applications/levels	0.74		
	5	Proportion of green technology transformation in traditional technology	0.81		
	6	Growth rate of number of green patent licenses	0.78		
	7	Proportion of green projects in the number of new product development projects	0.68		
	8	Number of papers published jointly by multi-agent cooperation	0.81		
	9	Number of joint application projects	0.72		
	10	Green technology achievement conversion rate	0.75		
The economic output of green technology innovation	11	New green products accounting for the total proportion of new products	0.77	70.21	0.896
	12	Export of new green products creating exchange rate	0.67		
	13	Percentage of green product sales revenue from new product sales	0.77		
	14	Market share of new green products	0.73		
	15	Government incentives and subsidies based on emission reductions	0.74		
	16	User acceptance of green technology products	0.80		
	17	Improved ROI on green innovation projects due to collaboration	0.76		
	18	Improvement in the success rate of new green product development through cooperation	0.74		
	19	Increased net profit margin due to cooperation	0.67		
The social effect of green technology innovation	20	Reduction rate of resource consumption per unit profit	0.69	75.58	0.870
	21	Rate of reduction in energy consumption per unit of profit	0.74		
	22	Growth rate of total labor productivity	0.78		
	23	Degree of improvement of public environmental preference and consciousness	0.81		
	24	Adoption of environmental management systems	0.66		
	25	Recycling rate of industrial waste	0.76		
	26	Carbon emissions per unit of profit	0.79		
	27	Discharge of three waste pollutants per unit profit	0.68		
	28	Forming national or industrial green technology standards	0.74		

**Table 6:** The second combined evaluation results.

Region	Value of the Second Combined Evaluation	Ranking	Region	Value of the Second Combined Evaluation	Ranking	Region	Value of the Second Combined Evaluation	Ranking
Beijing	0.7496	2	Zhejiang	0.5059	5	Hainan	0.2039	27
Tianjin	0.4039	7	Anhui	0.3180	12	Chongqing	0.2465	18
Hebei	0.2619	16	Fujian	0.3567	8	Sichuan	0.2917	15
Shanxi	0.2283	22	Jiangxi	0.2326	21	Guizhou	0.1924	28
Inner Mongolia	0.2161	24	Shandong	0.4698	6	Yunnan	0.2134	25
Liaoning	0.3190	11	Henan	0.3219	10	Shaanxi	0.3166	13
Jilin	0.2460	19	Hubei	0.3566	9	Gansu	0.2059	26
Heilongjiang	0.2469	17	Hunan	0.2985	14	Qinghai	0.1618	30
Shanghai	0.5282	4	Guangdong	0.7783	1	Ningxia	0.2386	20
Jiangsu	0.6368	3	Guangxi	0.2215	23	Xinjiang	0.1908	29
